# Does sequential examination under anaesthesia provide a reliable method to determine a management plan for unstable lateral compression pelvic ring injuries? a prospective study

**DOI:** 10.1007/s00590-023-03625-8

**Published:** 2023-07-05

**Authors:** Mostafa Ahmed Shawky, Ahmed Hazem Abdelazeem, Khaled Fawzy Abdel-Kader, Molham Mahmood Mohammad, Ahmad Hamdi Azzam

**Affiliations:** Pelvic Trauma and Arthroplasty Unit, Department of Orthopaedics and Traumatology, Kasr-AlAiny Hospital, Cairo University, 12 Al-Saraya Street, El Manial, Cairo, Egypt

**Keywords:** Fracture pelvis, Pelvic injuries, Lateral compression injuries, Examination under anaesthesia, Posterior-only fixation, Anterior**–**posterior fixation

## Abstract

**Purpose:**

To assess the reliability of sequential examination under anaesthesia (EUA) to determine pelvic instability and to evaluate radiological and functional outcomes in unstable lateral compression (LC) injuries.

**Methods:**

A prospective case series study was conducted from 2020 to 2022 at a university hospital on 43 cases with LC injuries that met the inclusion criteria. Sequential EUA was carried out in three steps. Posterior-only fixation or anterior–posterior fixation was done according to the algorithm. Each patient was followed up for at least 12 months, both radiologically and functionally.

**Results:**

Forty cases proved unstable and were fixed**.** None showed secondary displacement in the anterior–posterior fixation group. However, five cases (19.2%) of the posterior-only fixation group showed secondary displacement with a mean of 5.9 mm. Four cases of them had tetra-ramic injuries. There is a high tendency for secondary displacement at 14.5 mm or more preoperative displacement of the rami. Patients with secondary displacement showed comparable functional outcome scores to patients without secondary displacement. Posterior-only fixation showed shorter operative time, lesser radiological exposure, blood loss and iatrogenic nerve injury than anterior–posterior fixation.

**Conclusion:**

EUA is a reliable method to determine pelvic instability and management plan for LC fractures with unilateral anterior ring injury. Anterior–posterior fixation is needed if there is a tetra-ramic fracture or initial anterior ring displacement of 14.5 mm or more, irrespective of EUA.

## Introduction

Lateral compression (LC) fractures are the commonest pelvic fractures [1]. To date, there is no consensus on its management. This has been attributed to the heterogeneous spectrum of LC fractures, the inability to detect injured ligaments and the underestimation of the amount of displacement on static pelvic radiographs and computed tomography (CT) scans due to the natural recoil of the pelvis [2–6].

Since 2011, examination under anaesthesia (EUA) was advocated to assess the stability of pelvic injuries in general [7]. Moreover, in 2018, an algorithm using sequential EUA was suggested by Sagi et al. to guide a fixation strategy specifically for LC injuries. However, its drawbacks were its retrospective nature, lack of functional outcome and whether secondary displacement that occurred affected the functional outcome [2].

Our aim was to evaluate the reliability of sequential EUA for unstable LC injuries based on the algorithm suggested by Sagi et al. with minor modification. As a secondary outcome, we correlated the radiological to the functional outcomes to determine the impact of displacement on function.

## Materials and methods

### Ethical approval and consent to participate

Ethical approval was obtained from the institutional ethical committee review board (MD-214-2020) before collecting data. Informed consent was obtained from all individual participants included in the study.

### Patients and methodology

A prospective case series study was conducted in a university hospital from June 2020 to July 2022. We included high-energy trauma LC injuries: LC 1 injury with complete fracture sacrum (defined as extension of the fracture line to the posterior sacral cortex on axial CT cuts) and LC 2 and LC 3 injuries (including ipsilateral, contralateral or bilateral anterior ring injuries in any type). We excluded patients who did not complete a minimum of one-year follow-up, LC 1 injury with incomplete fracture sacrum**,** vertically unstable pelvic fractures, associated acetabular fractures, medically unfit patients for surgery, pregnancy, skeletally immature or geriatric (less than 18 years or more than 65 years) and neglected fractures more than three weeks.

**Fig. 1:**
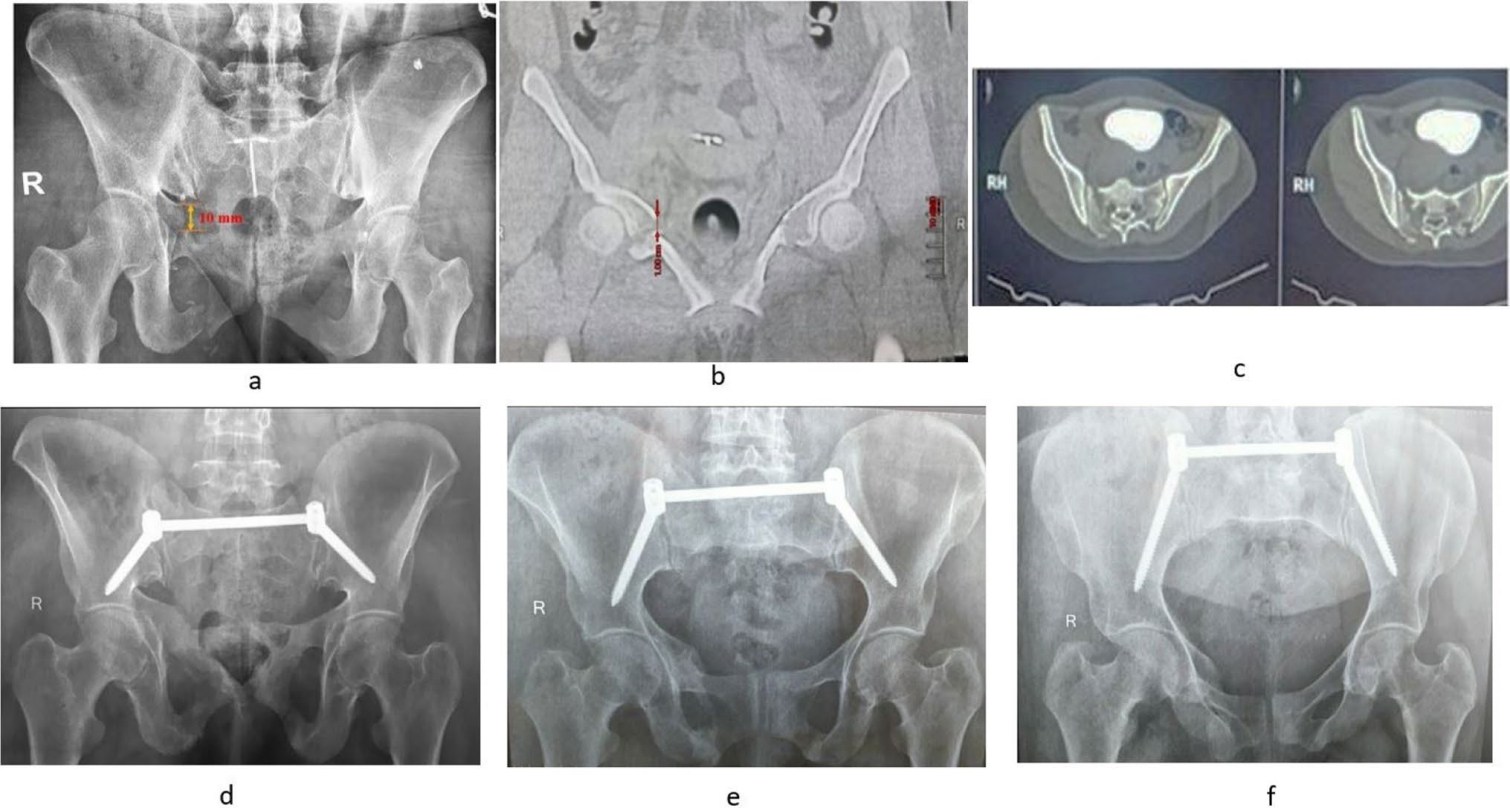
A LC 1 case with posterior-only fixation; measuring of initial displacement at the rami in preoperative AP X-ray (**a**) and CT (**b**), **c** axial CT cut, follow-up at one year is shown in the outlet (**d**), AP **e** and inlet **f** views. The patient healed with no secondary displacement

All patients were preoperatively evaluated clinically, radiologically and adequately prepared for the operation. All fractures were classified according to Young-Burgess and Day (for LC 2 injuries) [8, 9]. The distance was measured between each point on the fracture ends in the direction of maximum fracture displacement in the standard pelvic views (AP, inlet and outlet) with 100% magnification and reassessed using a CT scan (Fig. 1). The maximum value in any view was recorded and regarded as “the initial displacement.”

EUA was carried out by a single pelvic consultant in all cases. This was done supine on a radiolucent table. Management was done following a modified algorithm based on that proposed by Sagi et al. [2]. The modification was targeted for cases with bilateral anterior ring injury in which EUA was done bilaterally after posterior fixation. An anterior internal fixator was applied if instability was found at any side in any of the standard pelvic views.

**Fig. 2:**
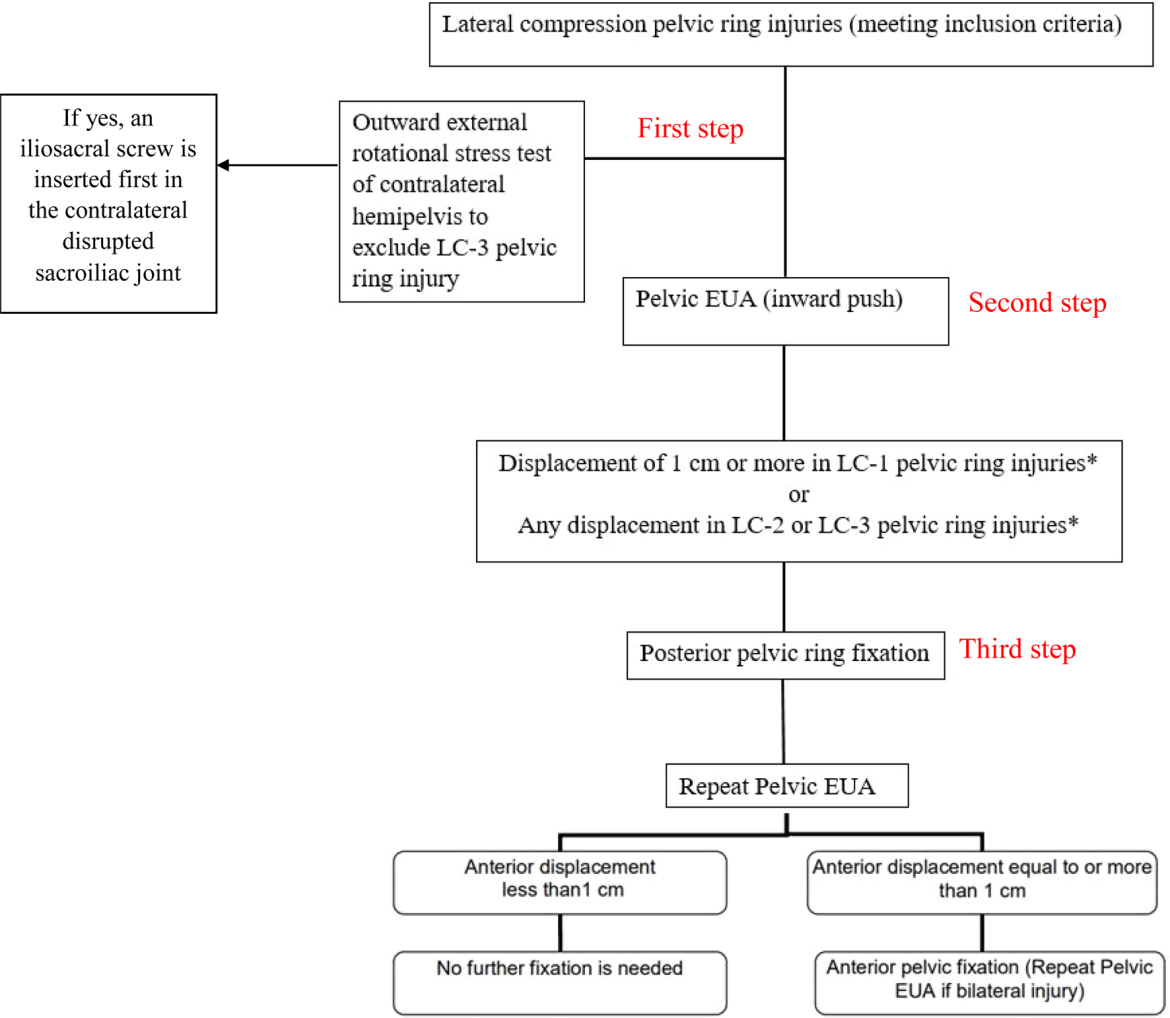
Sequential EUA algorithm for LC injuries

EUA was carried out in three steps as described in the algorithm (Fig. 2). The degree of displacement during EUA was measured using image intensifier by taking three static standard pelvic views and another exactly same three standard pelvic views after applying stress and calculating rami displacement in each view between fracture ends in direction of maximum displacement. By subtracting the rami displacement in the static view from its value in the stress view in each of AP, inlet and outlet, the resultant was the amount of displacement during stress. Decision was made according to maximum displacement in any view.

**Fig. 3:**
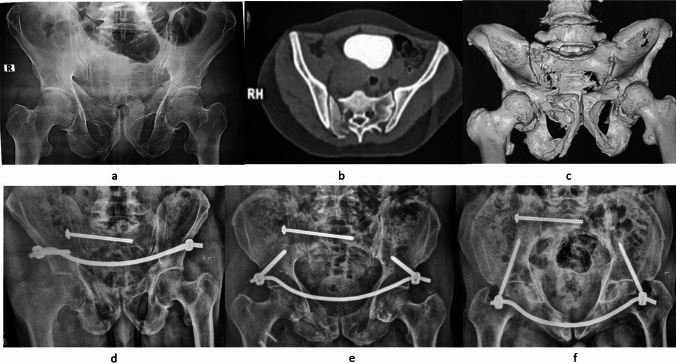
A LC 1 case with a tetra-ramic fracture. Anterior–posterior fixation was done. Preoperative AP X-ray **a**, axial CT **b** and 3D reconstruction **c** are shown. Follow-up at one year is shown in the outlet **d**, AP **e** and inlet **f** views. The patient healed with no secondary displacement

If instability was detected, posterior ring fixation was done first. In LC 1 with complete non-displaced simple sacral fracture, an iliosacral screw was done. In cases of LC 1 with a complete comminuted sacral fracture or sacral dysmorphism, a trans-ilial internal fixator (TIFI) was done. In LC 2 injuries, double anterior sacroiliac (SI) plating was done in Day 1 and 2, while iliosacral screw was done in Day 3 injuries. Posterior pelvic ring reduction was achieved in sacral fractures by closed reduction either by traction when inserting iliosacral screw or manipulation over TIFI screws. However, in LC 2 injuries, open reduction was achieved by traction combined with internal rotation, manipulation using a Schanz screw on T-handle and inserted in iliac crest, manipulation of the hemipelvis with a Farabeuf forceps or an asymmetric pelvic reduction clamp. An anterior internal fixator was applied if persistent instability was detected after posterior fixation (Fig. 3). No attempts of reduction were done on rami fractures.

Intraoperative data and post-operative complications were recorded. Post-operative standard pelvic views and CT were done to ensure the quality of reduction. Post-operatively, mobilisation in bed and toe-touch weight bearing were allowed till evidence of starting union on follow-up X-rays; then full-weight bearing was encouraged. Patients were followed up for at least 12 months radiologically and functionally. Radiologically, patients were assessed regarding union rate, time to union and occurrence of secondary displacement. Union was assessed in each visit radiologically in the standard pelvic views and clinically. It was defined as obliteration of the fracture line with callus formation in at least three cortices in rami fracture and clinically by absence of pain whether posteriorly or anteriorly and pain-free ambulation. Secondary displacement was defined as rotational or translational displacement of 5 mm or more from immediate post-operative radiographs in any of the standard pelvic views. The amount of secondary displacement was measured in each follow-up in reference to a known measured screw in the X-ray, followed-up each visit, and maximum displacement in any view was recorded. Functional outcome was assessed using the Majeed and pelvic outcome scores at one year [10, 11].

### Statistics

Data were coded and entered using the statistical package for the Social Sciences (SPSS) version 26 (IBM Corp., Armonk, NY, USA) and regarded *P* value <0.05 as statistical significance. Comparisons between quantitative data were made using unpaired *t* test and nonparametric Mann–Whitney test. For comparing categorical data, Chi-square (*χ*2) test was performed. A receiver operating characteristic (ROC) curve was done to identify a cut-off value.

## Results

**Fig. 4:**
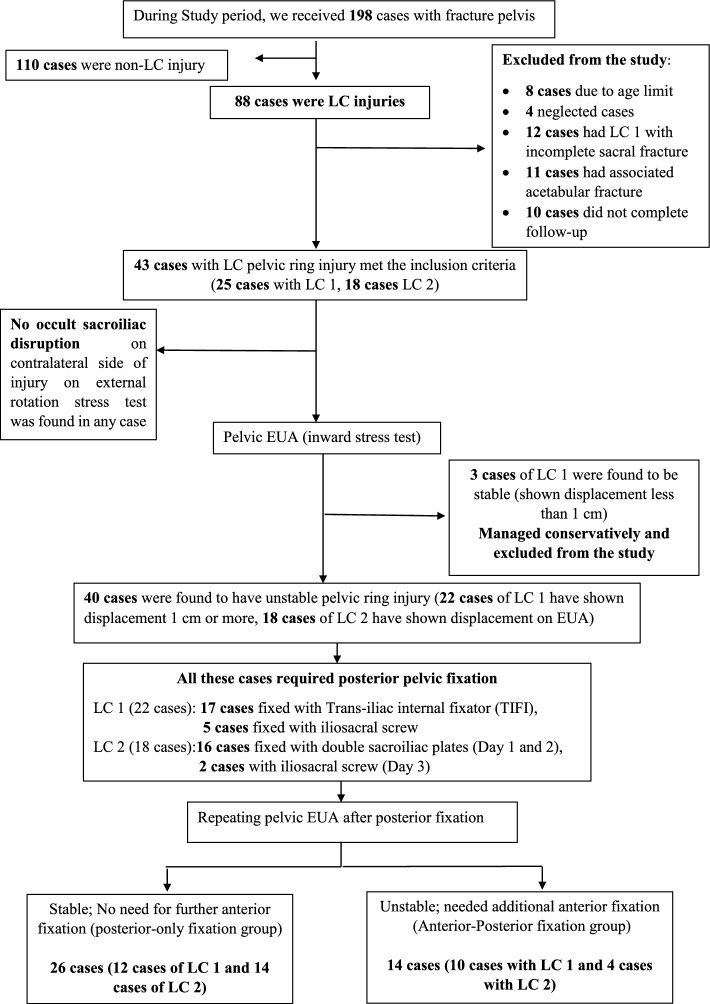
Flow chart of patients included in the study

During the study period, we received 198 cases of fracture pelvis. Only 43 cases with LC fracture met our inclusion criteria (Fig. 4). According to the algorithm, three cases (of LC 1 with complete sacral fracture) were stable, managed conservatively and excluded from the study. The remaining 40 cases were unstable and included in the study: 22 cases (55%) of LC 1, 18 cases (45%) of LC 2 and none of LC 3 injury. According to the algorithm, 26 cases (65%) underwent posterior-only fixation, while 14 cases (35%) underwent anterior–posterior fixation. Patients’ demographics are summarised in Table 1, operative data in Table 2 and post-operative outcomes and complications in Table 3.

**Table 1:** Summary of patients’ demographics of cases in this study

		Posterior-only fixation group	Anterior–posterior fixation group
Number of cases		26 cases (65%)	14 cases (35%)
Age (mean/ range)		32.4 years (18–54 years)	31.1 years (19–50 years)
Gender	Males	14 cases (53.8%)	6 cases (42.9%)
	Females	12 cases (46.2%)	8 cases (57.1%)
Mode of trauma	Road traffic accident	7 cases (26.9%)	6 cases (42.9%)
	Fall from height	13 cases (50%)	8 cases (57.1%)
	Motor car accident	6 cases (23.1%)	None
Associated non-pelvic fractures		13 cases (50%)	9 cases (64.3%)
Young-burgess classification		LC 1 (12 cases), 46.2%	LC 1 (10 cases), 71.4%
		LC 2 (14 cases), 53.8%	LC 2 (4 cases), 28.6%
Day classification for LC 2 injury	Day 1	9 cases (64.3%)	One case (25%)
	Day 2	3 cases (21.4%)	3 cases (75%)
	Day 3	2 cases (14.3%)	none
Pattern of anterior ring injury	Unilateral (ipsilateral)	17 cases (65.4%)	7 cases (50%)
	Unilateral (contralateral)	3 cases (11.5%)	3 cases (21.4%)
	Tetra-ramic	6 cases (23.1%)	4 cases (28.6%)

**Table 2:** Summary of operative data of cases in this study

	Posterior-only fixation group (26 cases)	Anterior–posterior fixation group (14 cases)
Time to surgery (days)	9.46 ± 3.39 (range 4–16)	9.21 ± 4.1 (range 3–15)
Mean initial displacement	12.35 mm (3–32 mm)	11 mm (4–20 mm)
*Implant for posterior fixation*		
TIFI	10 cases (38.5%)	7 cases (50%)
Iliosacral screw	4 cases (15.3%)	3 cases (21.4%)
Double SI plates	12 cases (46.2%)	4 cases (28.6%)
Operative time (min)	90 ± 29.9 min	143.21 ± 15.39 min
Radiological exposure(min)	2.66 ± 0.38 min	3.81 ± 0.36 min
*Blood loss (ml)*		
TIFI	149 ± 35.43 ml	417.14 ± 47.16 ml
Iliosacral screw	65 ± 12.9 ml	366.67 ± 76.38 ml
Double SI plates	545.83 ± 58.23 ml	537.5 ± 110.87 ml

**Table 3:** Summary of radiological and functional outcomes and complications of cases in this study

	Posterior-only fixation group (26 cases)	Anterior–posterior fixation group (14 cases)
Secondary displacement	5 cases (19.2%); (2 cases of LC 1, 3 cases of LC 2)	None
Mean secondary displacement	5.9 ± 0.7 mm (range from 5–6.9 mm)	None
The pattern of anterior ring injury in displaced cases	4 cases (80%): tetra-ramic injury One case (20%): unilateral (contralateral injury)	None
Mean initial displacement at the anterior ring in displaced cases	19.8 ± 10.4 mm (range 10–32 mm)	None
Time to union	13 ± 2.14 weeks	13.14 ± 2.32 weeks
Majeed score at one year	92.73 ± 3.38 ( 82–97)	90.07 ± 4.38 (82–96)
Pelvic outcome score at one year	33.54 ± 2.35 (29–37)	33.29 ± 2.09 (28–36)
Time to full-weight bearing	13.38 ± 1.75 weeks	13.29 ± 2.27 weeks
Follow-up period	13.08 ± 1.65 months	13.21 ± 2.42 months
Wound infection	None	2 cases
Iatrogenic nerve injury	None	4 cases

Posterior-only fixation group showed shorter operative time, lesser radiological exposure and blood loss (when using TIFI or iliosacral screw) than anterior–posterior fixation group, with a statistically significant difference (*P* value <0.001 for all parameters). However, cases fixed with double anterior sacroiliac plating showed comparable blood loss in both study groups, with no statistically significant difference (*P* value = 0.846).

**Fig. 5:**
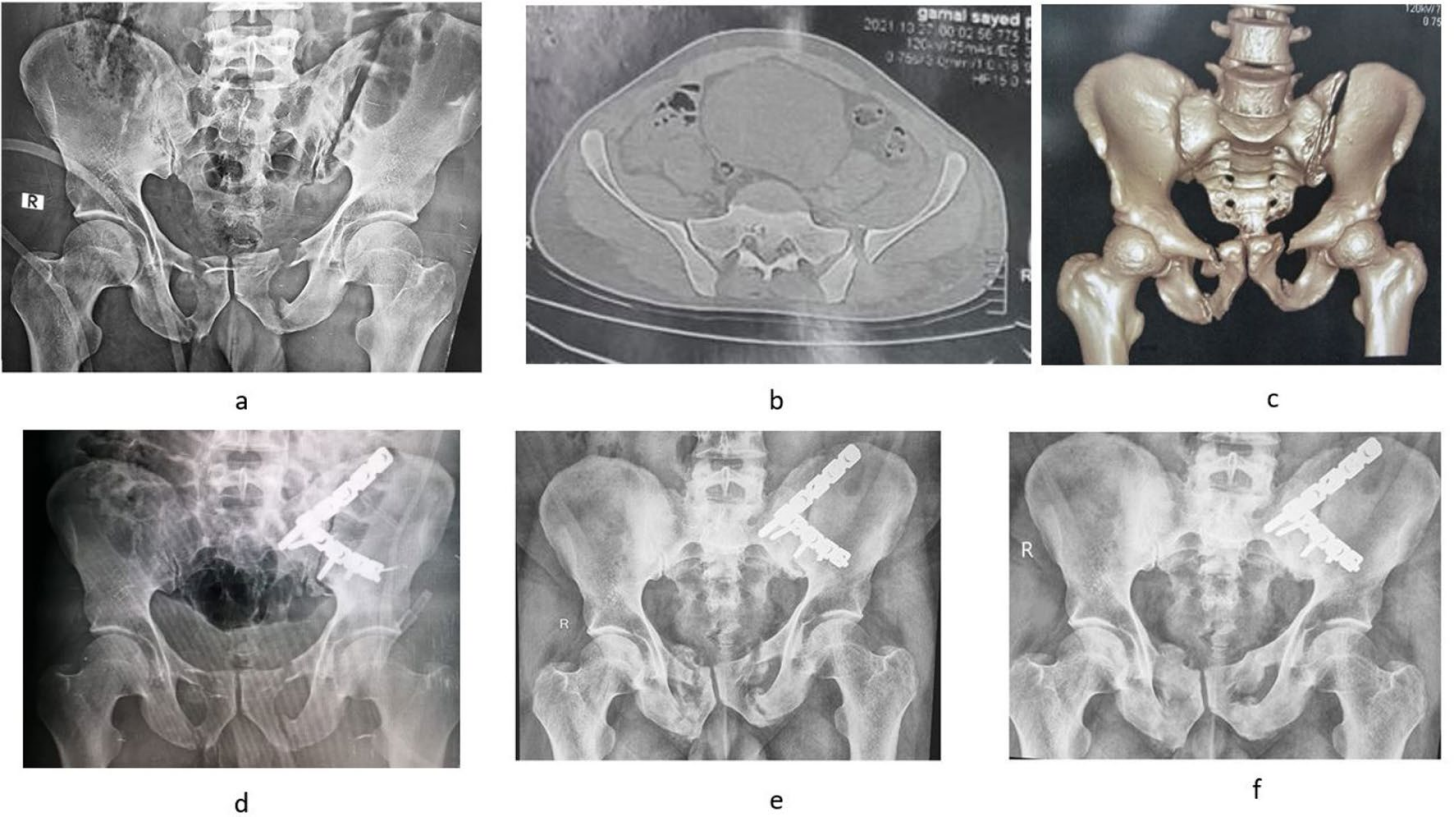
A LC 2 case (Day 1) with tetra-ramic fracture. Posterior-only fixation was done. Preoperative AP X-ray (**a**), axial CT (**b**) and 3D reconstruction (**c**). Immediate post-operative AP X-ray is shown (**d**). Fracture showed maximum secondary displacement of 5.8 mm in AP view on eight-week follow-up (**e**). The fracture healed with no further displacement; follow-up AP view at one year (**f**)

All cases had attained union with no statistically significant difference between both groups as regards time to union (*P* value = 0.846). None of the cases has shown secondary displacement in anterior–posterior fixation group, while almost 20% of posterior-only fixation group (five cases) have shown secondary displacement at the rami occurred within the first 12 weeks post-operatively. Four of the five cases had a tetra-ramic injury (Fig. 5). Patients with tetra-ramic injury in posterior-only fixation group showed a higher rate of secondary displacement representing 67% of cases with tetra-ramic fracture, which was statistically significant (*P* value = 0.009). In posterior-only fixation group, we noticed that patients who showed secondary displacement had a statistically significant higher initial anterior ring displacement than cases that did not show further secondary displacement on follow-up (19.8 mm and 10.6 mm, respectively). Using statistical analysis (ROC curve), we identified that at an initial anterior ring displacement of 14.5 mm or higher, secondary displacement will occur in cases fixed posterior only (*P* value <0.001), with the area under the curve = 0.833 and 95% confidence interval = [0.65, 1.01], a sensitivity of 60% and specificity of 85.7%.

Functionally, there was no statistically significant difference between both groups as regards Majeed score and pelvic outcome score at one year (*P* value is 0.276 and 0.738, respectively). In posterior-only fixation group, there was no statistically significant difference between patients who showed secondary displacement and those who did not show secondary displacement as regards Majeed and pelvic outcome scores at one year (*P* value is 0.841 and 0.637, respectively).

Concerning iatrogenic nerve injury, four cases (28.6%) in anterior–posterior fixation group had post-operative lateral cutaneous nerve of the thigh (LCNT) injury related to anterior internal fixator application, while none showed any iatrogenic nerve injury in posterior-only fixation group, with a statistically significant difference (*P* value = 0.011); three cases had a unilateral LCNT injury, and one had a bilateral injury, all resolved within six months. Two cases in anterior–posterior fixation group had post-operative wound infection compared to none in posterior-only fixation group, with no statistically significant difference (*P* value = 0.117). One case had a superficial infection over TIFI screws incisions which was resolved by repeated dressing and antibiotics. The other case had a deep infection at the lateral window of the ilioinguinal incision, which was treated by a single session of surgical debridement and retention of implants.

## Discussion

LC 1 fractures were previously considered stable injuries [1]. However, it is now settled that LC 1 fractures involve a heterogeneous spectrum of injuries [6]. The non-operative management of LC 1 injury yielded poor results, especially in complete sacral with a tetra-ramic fracture [12]. Henderson demonstrated that LC 1 fractures with more than 1 cm of initial sacral displacement had poorer clinical outcomes when treated conservatively [13]. Beckmann et al. reported low agreement among OTA surgeons, with no overall difference in the decision for operative stabilisation for heterogeneous cases of LC 1 [14]. The patients with 10 mm or more of displacement on lateral stress radiography (LSR) were more likely to have difficulty mobilizing secondary to pain, undergo delayed operative fixation, have longer hospital stays and use more opioids [15]. In our study, three (12%) out of 25 LC 1 cases were stable, according to EUA. In contrast, 22 cases (88%) were unstable. All cases attained union with no secondary displacement except for two LC 1 cases (out of 12 cases) in posterior-only fixation group which showed minimal displacement but satisfactory functional outcomes. We noticed that EUA revealed a greater proportion of LC 1 to be unstable, which had been previously thought to be stable on static imaging. Moreover, we followed up the three stable cases, with no secondary displacement noticed. These findings proved the reliability of the EUA algorithm to detect instability in LC 1 injuries which was consistent with Whiting et al. and Tucker et al. Whiting et al. reported on the effectiveness of a negative EUA finding in predicting radiographic union without displacement [16]. Tucker et al. concluded that EUA increased agreement among experienced pelvic surgeons to assess pelvic stability and the need for operation [17].

LC 2 injuries are more inherently unstable than LC 1 injuries. The gold standard for the management of this type of injury is fixation. However, some authors reported that rigid isolated posterior fixation has the same functional outcomes, with the advantages of lesser blood loss and operative time when compared to combined posterior and anterior fixation [18–20]. On the other side, other authors emphasized the importance of fixation of all pelvic ring’s injured elements for better anatomical results [21]. In our study, we encountered 18 LC 2 cases; all were unstable and required fixation. Three cases in posterior-only fixation group had a tetra-ramic injury and showed secondary displacement with acceptable functional outcome scores at one year. We noticed that

LC 2 injuries with tetra-ramic injury are highly unstable and require combined posterior and anterior fixation. This was consistent with Weaver et al. [5] who concluded that tetra-ramic fractures in LC injuries had a higher rate of initial displacement and signified more unstable injury. Although more unstable, we found a higher rate of posterior-only fixation in LC 2 injuries (77.8%) compared to LC 1 injuries (54.5%), with no secondary displacement and excellent functional outcomes. This is probably because most cases were fixed with double anterior SI plating (85.7%) in posterior-only fixation group which is biomechanically superior to other methods used for LC 1 fixation, obviating the need for anterior fixation. However, this applies to cases with unilateral anterior ring injury only.

A recent study [22] reported that around 11 (52%) out of 21 LC cases treated with posterior-only fixation showed secondary displacement, all of which had either oblique or comminuted rami fracture, irrespective of completeness of sacral fracture. They recommended EUA in these patterns and combined fixation in such cases. However, it was a retrospective study and didn’t use the sequential EUA algorithm in management and lacked the methodology upon which the decision was made for posterior-only or anterior–posterior fixation. It is unclear whether using the algorithm would have detected further instability in the anterior ring, thus avoiding secondary displacement.

Recently, Parry et al. and other authors recommended the use of emergency department (ED) stress radiography in LC 1 cases as an effective and reliable method for detecting occult instability as compared to EUA and to Beckmann scoring system [23] of pelvic instability with excellent functional and radiological outcomes, thus reliably detecting cases which need surgery [24–28]. Moreover, LSR in ED has been advocated by Parry et al. [26–28]. The advantages of LSR over EUA are no need for anaesthesia or operative room, obviating radiological exposure to the examiner and the standardization of force applied to the pelvis, with the ability to make formal measurements of displacement on radiographs that cannot be easily performed intraoperatively with fluoroscopy [27]. Parry et al. demonstrated that the sensitivity and specificity of the LSR to identify unstable LC1 pelvic injuries as determined by the EUA were both 100% and demonstrated 100% correlation with EUA [28]. However, LSR is limited by the patient’s tolerance to the lateral decubitus and it cannot demonstrate an occult sacroiliac disruption for LC 3 injury although LC 3 does not occur in a negative-stress (stable) patient on LSR [27, 28].

In this study, sequential EUA led to union in LC cases containing unilateral rami fracture (whether ipsilateral or contralateral) without secondary displacement during the follow-up. However, in tera-ramic fractures in posterior-only fixation group, there was a higher tendency for secondary displacement. These findings were consistent with Sagi et al. [2]. This may be due to some force (during EUA in tetra-ramic fracture) being lost at the contralateral fracture of the anterior ring, leading to the inaccurate displacement of the rami at the examined side and a false small amount of displacement, which was interpreted as falsely stable after posterior fixation. Yet, functional outcome scores of cases that showed secondary displacement were comparable to those that did not show secondary displacement. However, it is better to add anterior fixation in these cases to obtain rigid fixation, irrespective of EUA.

We identified a cut-off value of initial displacement at the anterior ring at which there is a higher tendency for secondary displacement when the patient is fixed posteriorly only, that is, at 14.5 mm or more. Thus, we recommend combined fixation in these cases, irrespective of EUA. However, this cut-off value must be studied for accuracy on a larger sample size.

The strengths of this study include that it is the first prospective study, to the best of our knowledge, which evaluates sequential EUA in LC injuries regarding functional and radiological outcomes and intraoperative parameters between study groups. Moreover, it identifies a cut-off value of initial displacement of the anterior ring (≥ 14.5 mm) at which there is a higher tendency for secondary displacement with posterior-only fixation.

The limitations of this study include a small cohort of patients, a diversity of implants used, and arbitrarily using 1 cm as the cut-off value during EUA to detect instability, as used previously by Sagi et al. [2]. The disadvantages of EUA include the need for an operating room, anaesthesia and the vector of force during EUA, the subjective measurement and interpretation of EUA which depends on the operating surgeon. However, this was overcome in our study as a single surgeon did EUA for all cases. One of the limitations is that we did not encounter any LC 3 injury although it was in the inclusion criteria. We recommend further studies to assess EUA on a cohort of LC 3 cases for a better evaluation of reliability of EUA algorithm in LC 3 injuries.

Another limitation of the study is using completeness of sacral fracture in LC 1 injuries as a determinant for inclusion. Hadeed et al. concluded that completeness of sacral fracture in CT axial cuts in cases with minimally displaced LC 1 injuries had weak inter-observer reliability and sacral fractures that were considered incomplete by all surgeons did have occult instability [29]. Thus, we recommend further studies on LC 1 injuries using EUA irrespective of the completeness of sacral fracture to detect instability.

This study focused primarily on testing the reliability of EUA in determining management plan for LC fractures in terms of radiological and functional outcomes; however, early post-operative data, pain scores and opioid use remain an important outcome which was not addressed in this study. Some authors concluded that anterior–posterior fixation in LC 1 injuries reduced in-patient opioid use and an increased number of patients who could clear physiotherapy and discharge home compared to posterior-only fixation [30].

To conclude, sequential EUA is a reliable method to determine intraoperative pelvic instability and management plan for LC injuries containing unilateral rami fracture. In cases with tetra-ramic injury or initial anterior ring displacement of 14.5 mm or more, combined posterior and anterior fixation is needed, irrespective of EUA, to obtain rigid fixation. Further studies on a larger sample size and longer follow-up periods addressing both functional and radiological outcomes need to be conducted to determine accurately the preoperative cut-off displacement in the anterior ring, which affects secondary displacement in LC injuries. Biomechanical studies need to be conducted to determine the amount of force and identification of cut-off value which signifies pelvic instability during EUA.

## Data Availability

Yes.
